# Stressful Symmetry: Bilateral Bell’s Palsy Potentially Induced by Extreme Stress

**DOI:** 10.7759/cureus.82704

**Published:** 2025-04-21

**Authors:** Hassan Ahmed, Maddiah Mazahr, Jahanzeb Rehan

**Affiliations:** 1 Internal Medicine, Queen Elizabeth Hospital King's Lynn NHS Foundation Trust, King's Lynn, GBR; 2 Internal Medicine, King's Mill Hospital, Nottingham, GBR; 3 Stroke Medicine, King's Mill Hospital, Nottingham, GBR

**Keywords:** acute stress illness, bell's palsy, facial asymmetry, facial nerve paralysis, idiopathic bilateral simultaneous facial nerve palsy

## Abstract

Facial nerve palsy (FNP) of idiopathic origin is commonly called Bell's palsy. Bilateral FNP represents an exceptionally rare subset of all Bell's palsy cases. It may manifest either synchronously or sequentially, with one side initially affected, followed by involvement of the opposite side within the initial 30 days of symptom onset. Common etiologies of FNP encompass infections such as infectious mononucleosis, influenza, Lyme disease, and meningitis. Non-infectious causes comprise neoplasms, multiple sclerosis, and Guillain-Barre syndrome. In this particular case, our patient experienced extreme stress prior to the onset of symptoms. Following the exclusion of common causes of secondary FNP, the patient was diagnosed with bilateral Bell's palsy. We have explored the consideration that extreme stress may serve as a precipitating factor for bilateral Bell's palsy.

## Introduction

The typical presentation of facial palsy is unilateral, manifesting in a lower motor neuron pattern. The predominant presentation involves the sudden onset of facial nerve palsy (FNP), typically evolving over hours and frequently resolving spontaneously within months. The most common cause is idiopathic, commonly known as Bell’s Palsy [1]. In the UK, the incidence of Bell's Palsy is 37.7 per 100,000 population, constituting approximately 60% of all reported cases of FNP [2].

In contrast, bilateral FNP remains a rare occurrence, representing approximately 2% of all cases of FNP [3]. Bilateral FNP is characterized by facial paralysis starting on one side initially, followed by involvement of the contralateral side. Alternatively, it may present as the simultaneous onset of bilateral facial paralysis [4]. The causes of bilateral FNP often involve significant underlying systemic diseases; however, Bell's palsy (idiopathic) accounts for less than 20% of cases of bilateral FNP [3].

This case study focuses on a male in his early 70s who presented with unilateral facial palsy, followed by facial palsy affecting the contralateral side. The gentleman has a history of adequately controlled hypertension through diet and does not have any newly diagnosed systemic illnesses or pending investigations. He reported experiencing extreme stress prior to the onset of his symptoms. We further discuss the differential diagnoses, diagnostic approach, and management of the disease.

## Case presentation

A male in his early 70s, with a background of diet-controlled hypertension, noticed some unilateral facial asymmetry. This progressed rapidly, and he had complete paralysis of the right side of his face by the end of the day. He went to the emergency department (ED), where he was diagnosed and managed for Bell’s palsy and discharged home with a 10-day course of prednisolone.

At approximately three weeks after the initial onset of right-sided facial paralysis, he developed left-sided facial weakness, which progressed rapidly and led to complete paralysis of the left side; this was in addition to the ongoing right-sided paralysis. During the three weeks, he did not experience any improvement in his symptoms involving the right side, and he presented to the ED then with bilateral lower motor neuron facial palsy.

The main symptoms reported by the patient were a throbbing headache located behind both ears and drooping of the angle of the mouth on both sides. He also reported an altered taste sensation and dry eyes. Symptoms were more pronounced on the right side, despite it being affected three weeks earlier than the left.

One thing of note is that the patient reported significant stress for a few weeks before this illness. In his own words, “I have never been this stressed in my life." He attributed this stress to frequent heated arguments within his family, which had been particularly intense lately. While he had coped well with workplace pressure before retiring, he struggled to handle the family conflicts. He rated his stress level as a "9 out of 10" in severity. His circumstances improved after getting admitted to the hospital, and he reported he did not feel as stressed during his inpatient stay. Due to this improvement, we were unable to utilize standardized stress scales for assessment.

The patient denied any history of trauma, a preceding viral illness, travel history, or weight loss.

On examination, he was alert, oriented, and comfortable. He had a blood pressure of 150/89, a heart rate of 95, and a temperature of 36.4 °C. Upon auscultation, the chest was clear, and the abdomen was soft and non-tender with no organomegaly of note. There was no evidence of lumps, swellings, or edema.

When asked to close his eyes, he was unable to close the eyelids fully, and the sclera remained visible. He also had drooping of the angle of the mouth bilaterally. These findings were more pronounced on the right side. He was unable to puff up his cheeks, and drinking any liquid caused it to drool down from the corners of his mouth. The patient was able to lift his eyebrows. Examination of other cranial nerves was normal. There was no other neurology on examination of peripheral nerves, and deep tendon reflexes were normal.

Initial investigations involved chest X-ray and CT head, which were unremarkable. During the second admission to the hospital, a comprehensive set of investigations was carried out. CT chest, abdomen, and pelvis with contrast did not show any pathology. The test results showed CRP at 0.9 mg/L, hemoglobin at 15.8 g/dL, platelets at 464 x 10³/µL, and a white cell count of 5.7 x 10³/µL. Urea was 5.9 mmol/L, creatinine 80 µmol/L, sodium 143 mmol/L, potassium 4.4 mmol/L, and chloride 112 mmol/L. Magnesium was 0.92 mmol/L, HbA1c 5.8%, angiotensin-converting enzyme levels 39 IU/L, alkaline phosphatase of 58 U/L, ALT at 15 U/L, bilirubin at 6 µmol/L, and albumin at 40 g/L, which were all essentially within normal limits. Serologies, including borrelia serology, HIV, anti-ganglioside antibody screen, ANA, ANCA, and ENA, were all negative.

A lumbar puncture was performed, which was unremarkable with normal protein levels and without any growth on culture.

**Figure 1:**
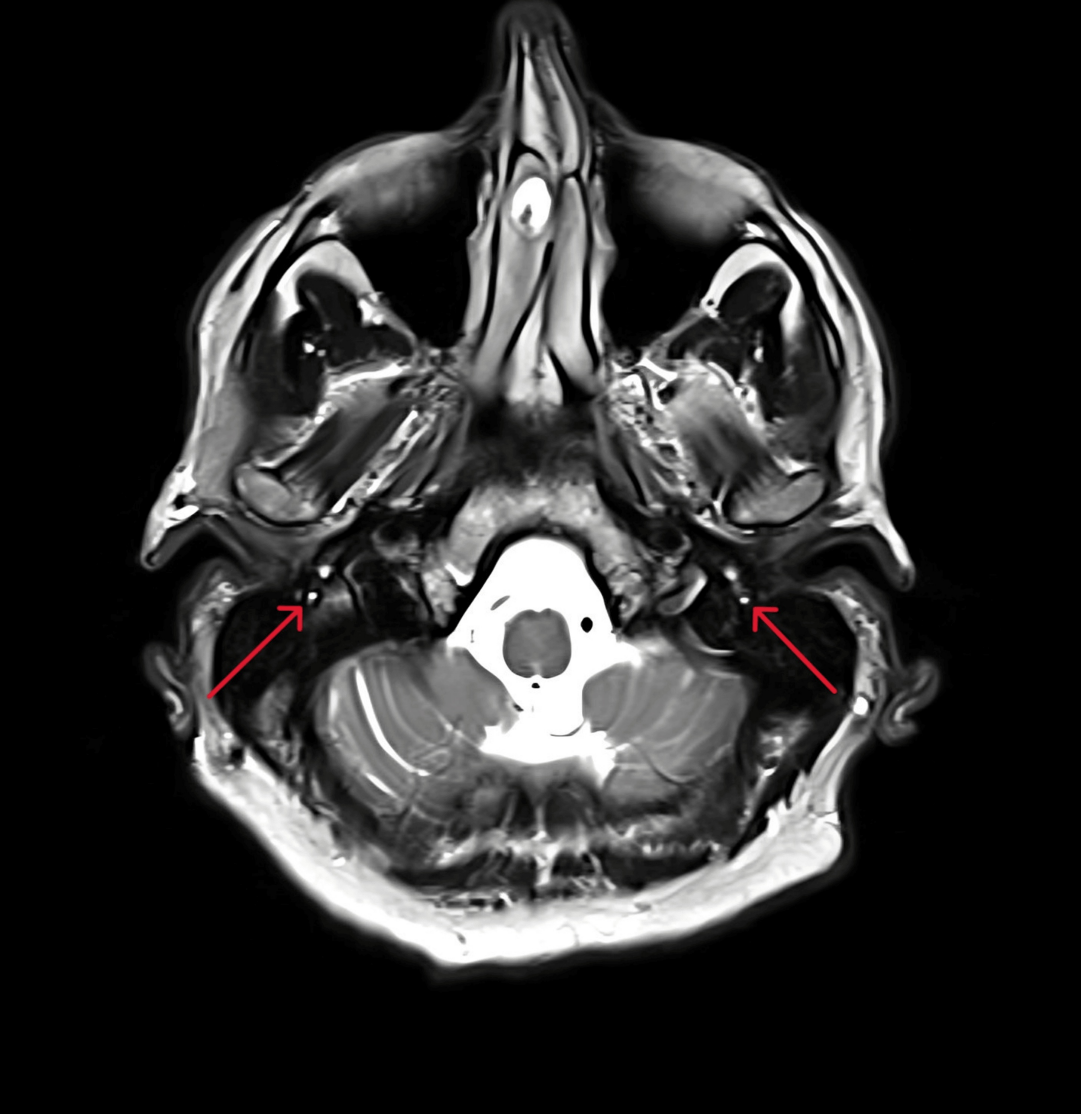
Contrast-enhanced MRI scan displaying abnormally high T2 signal of bilateral facial nerves, indicated by the red arrows.

An MRI with contrast was performed, which showed a bilateral symmetrical uniform high T2 signal (Figure 1) and post-contrast enhancement in the tympanic and mastoid parts of both facial nerves. There was no evidence of a nerve sheath tumour.

After ruling out all the potential causes of bilateral FNP and with suggestive MRI features, this patient was diagnosed with bilateral idiopathic FNP (bilateral Bell's palsy). After a period of observation, the patient remained stable and was discharged home with an outpatient neurology follow-up.

During the follow-up telephone interview two months later, the patient reported that the paralysis on his left side had completely resolved, but he continued to experience symptoms on his right side. Since discharge, there has only been a slight improvement in the function of his right eyelid, but he has not regained any function in the right-sided facial muscles. In addition, he mentioned difficulty with food getting stuck in the right buccal space, although the issue with drooling from the corners of his mouth has been resolved bilaterally.

## Discussion

A critical challenge remains for physicians in establishing the etiology of bilateral FNP. This condition is often caused by an underlying systemic illness, with a vast array of potential causes including neurological, metabolic, autoimmune, vascular, idiopathic, congenital, neoplastic, trauma, or infectious factors. Many of the potential causes of bilateral FNP are severe and could be life-threatening; therefore, a thorough investigation is warranted to guide medical management [5].

To aid in the diagnosis, a comprehensive history is required, with particular emphasis on the time of onset, recent travel, preceding viral illnesses, otological symptoms, and neurological symptoms. This should be followed by a detailed clinical examination, with a focus on neurological assessment. Combining a detailed history with clinical examination can help identify the cause and guide medical management.

After taking the history, the next step is to consider differential diagnosis and send relevant narrow down the underlying etiology. The most common causes of bilateral FNP reported across the literature are Lyme disease, Guillain-Barré syndrome (GBS), sarcoidosis, and trauma [5]. Table 1 presents a list of possible etiologies, but it is in no way exhaustive.

**Table 1:** Common causes of bilateral facial nerve palsy Reference: [5]

Cause of disease	Associated condition
Trauma	Skull fractures
	Parotid surgery
	Mastoid surgery
Infection	Post-influenza
	Infectious mononucleosis
	HIV infection
	Lyme disease, Banwarth's syndrome
	Brainstem encephalitis
	Poliomyelitis
	HTLV-1 infection
	Syphilis
	Guillain–Barre syndrome
Metabolic	Diabetes
	Acute porphyria
Neoplastic	Acute leukaemia
	Acoustic neuroma
Autoimmune	Sarcoidosis
	Amyloidosis
Neurological	Multiple sclerosis
	Pseudobulbar and bulbar palsy
	Parkinson's disease
Idiopathic	Bell's palsy

Life-threatening causes need to be ruled out immediately. Severe, life-threatening trauma causing bilateral temporal bone fractures is known to lead to bilateral FNP. In this case, there was no history or evidence of trauma, which was confirmed by head imaging.

A rare, potentially life-threatening disease to rule out is GBS, an acute post-inflammatory polyneuropathy often preceded by illness commonly caused by *Campylobacter jejuni*, Epstein-Barr virus, or even influenza. In this instance, there was a low suspicion of GBS, as the patient did not exhibit any signs of ascending limb weakness or areflexia, which are characteristic of GBS. In addition, the patient did not report prodromal illness prior to developing facial palsy. These findings, in conjunction with negative anti-ganglioside antibody test results, led to the exclusion of GBS as an underlying aetiology [6]. Multiple sclerosis was not considered a likely diagnosis due to the absence of characteristic neurological findings on the MRI scan and the absence of oligoclonal bands or suggestive findings on lumbar puncture.

Common infectious causes include Lyme disease, a tick-borne illness caused by *Borrelia burgdorferi *spirochetes. The incidence of Lyme disease in the UK is estimated to be around 2000-3000 cases per year, with the highest number of cases in the south of England and the Scottish Highlands [7]. In this case, the patient denied recent travel to woodland areas or hikes and did not exhibit characteristic signs of Lyme disease [8]. Borrelia serology testing yielded negative results. Other infections, such as meningitis, were not highly suspected due to the absence of characteristic symptoms; however, this was excluded following unremarkable lumbar puncture results and normal CT head findings.

Certain neoplasms are known to cause cranial nerve palsies both as part of paraneoplastic syndromes or direct infiltration of the nerves [9]. It is important to exclude these on imaging and blood tests, as we did for this patient based on CT head, MRI head, and CT thorax, abdomen, and pelvis. We did not investigate leukaemia following normal full blood count (FBC) blood tests and based on clinical history.

The aetiology of Bell's palsy is generally believed to be the result of a viral inflammatory immune response, likely following the reactivation of a dormant virus. In this particular case, the patient experienced extreme stress prior to the initial presentation, and studies have demonstrated that the reactivation of a dormant virus during a period of immunosuppression secondary to high levels of stress may precipitate Bell's palsy [10]. At present, there are no reported cases of stress precipitating bilateral Bell’s palsy. However, in this case, stress may likely be the precipitant of bilateral FNP.

It is noteworthy that the synchronous onset of bilateral FNP is often associated with severe systemic illness; however, in this patient, the initial presentation was right-sided facial paralysis, followed by left-sided facial paralysis within three weeks, with a strong preceding history of stress. This difference in presentation may suggest how stress may be the cause of initial unilateral facial paralysis followed by bilateral FNP. It is essential for clinicians to evaluate psychosocial factors when assessing a patient with bilateral FNP. This may prompt psychosocial intervention, stress management, and counselling to aid in the individual's recovery. It is important to emphasize that patients presenting with unilateral FNP may benefit from a focused history of stress to gauge the severity of stress and anxiety, which can then aid in recommending appropriate management [11].

## Conclusions

Unilateral facial palsies are typically idiopathic, whereas bilateral facial palsies often suggest a potentially significant underlying systemic disease. After ruling out common causes of bilateral FNP, this patient received a diagnosis of idiopathic bilateral facial nerve palsy - bilateral Bell's palsy. A thorough history revealed that the patient experienced an unusually high level of stress prior to the onset of facial paralysis. Therefore, evaluating stress levels is essential when managing bilateral FNP, as stress may potentially be a precipitating factor.
